# Gestational diabetes severity stratification during pregnancy: role of plasma oleic acid as a possible early marker

**DOI:** 10.1007/s00592-025-02487-2

**Published:** 2025-04-01

**Authors:** Chiara M. Soldavini, Gabriele Piuri, Paola A. Corsetto, Irma Colombo, Veronica Resi, Stefania Zava, Gabriele Rossi, Enrico Ferrazzi, Angela M. Rizzo

**Affiliations:** 1 Obstetrics Unit, Department of Woman Child and Newborn, Fondazione IRCCS Ca’ Granda, Ospedale Maggiore Policlinico, 20122 Milan, Italy; 2 Department of Pharmacological and Biomolecular Sciences “Rodolfo Paoletti”, University of Milan, 20134 Milan, Italy; 3 Endocrinology Unit, Fondazione IRCCS Ca’ Granda Ospedale Maggiore Policlinico, 20122 Milan, Italy; 4 Research Center in Clinical and Translational Maternal Fetal and Neonatal Medicine, Department of Clinical and Community Sciences, University of Milan, 20100 Milan, Italy

**Keywords:** Gestational diabetes, Lipidomics, Oleic acid, Fatty acid, Pregnancy inflammation, Biomarker

## Abstract

Normal pregnancy is characterized by changes in lipid metabolism with significant implications for the health of both mother and offspring. When these changes develop into maternal dyslipidemia, a significant association with adverse pregnancy outcomes has been observed, including the development of gestational diabetes (GD), modulation of the inflammatory response, and excessive fetal growth. In the present study, we performed a lipidomic assessment of patients at GD diagnosis (24–28 weeks of gestation) and 12 weeks after diagnosis. We found higher levels of esterified oleic acid in plasma at the time of GD diagnosis in women who subsequently required pharmacological therapy to control blood glucose levels compared to those who did not require additional treatment, suggesting that the measurement of plasma oleic acid might be an additional tool for the early identification of patients with a more severe form of gestational diabetes. Moreover, plasma oleic acid levels showed a positive correlation with fetal growth in the context of adequate glycemic control, supporting a metabolic dysregulation of other pathways whose identification could help clinicians to discriminate different cases within the spectrum of severity of the disease. Finally, the correlation between plasma oleic acid and circulating BAFF levels at the time of diagnosis and 12 weeks later adds a possible mechanism to support the pro-inflammatory and pro-diabetic state in the metabolic set of GD. Overall, these findings strongly support the role of plasma oleic acid as a possible early marker for GD severity stratification during pregnancy.

## Introduction

Insulin resistance is the well-known mechanism in physiological pregnancy to redistribute fuels from the maternal compartment to ensure the fetal needs for metabolism and growth. Insulin resistance is involved in two principal sites, liver and peripheral tissue. It has been clearly shown in animal and human studies the reduced ability of insulin to modulate glucose uptake especially during the second half of pregnancy [1]. The liver seems as well affected by this defect of insulin action because hepatic glucose reduction is not complete and is delayed especially in obese non-diabetic subjects towards the term of pregnancy [2]. Moreover, insulin has been shown to be defective in pregnancy in reducing levels of free fatty acids (FFAs) indicating that insulin resistance observed in physiological conditions is a mechanism related not only to the glucose metabolism.

According to the “Modified Perdesen’s hypothesis”, all the fuels, carbohydrates, aminoacids and lipids, are diverted to the fetus for the energy-balance programming. As outlined by Freinkel: “Fetal growth and overgrowth is a function of multiple nutritional factors in addition to glucose” [3, 4]. In spite of this strong biological affirmation, only glucose has been clearly focused to evaluate and to establish the levels of insulin resistance especially in terms of diagnosis of pathological conditions as gestational diabetes (GD). In a similar manner, glucose has been used as the unique standard to evaluate the severity of diabetic conditions in pregnancy, excluding the possibility of introducing new diagnostic systems and therapeutic approaches.

Our preliminary results indicate that increased levels of triglycerides (TAGs) are associated with cases of GD that require a pharmacological approach [5]. Maternal dyslipidemia can affect the nutritional intrauterine environment and embryonic metabolism and development leading to adverse pregnancy outcomes [6]. Elevated TAGs concentrations during pregnancy have been reported to correlate with the development of GD, preeclampsia, intrahepatic cholestasis, large for gestational age (LGA), and macrosomia [7]. Changes in the fatty acid (FA) profiles of erythrocyte membranes and plasma contribute to insulin resistance and altered insulin secretion; since different FFAs have opposite effects on circulating inflammatory cytokine levels, including interleukin (IL)-6, IL-8, tumor necrosis factor-alpha (TNF-α) and resistin [8], these effects may be either direct or indirect, depending on the modulation of the inflammatory response. Moreover, increased levels of TAGs can accumulate in liver cells, amplifying the physiological mechanism of gestational insulin resistance. Therefore, an increased availability of TAGs can start a vicious circle.

In this context, a lipidomic approach could help to characterize and quantify lipid species that might influence the metabolic conditions of GD in disease progression [9, 10]. Recently, lipidomic has been explored as an additional tool that may improve the prediction of GD by detecting early metabolic dysregulation to limit short and long-term complications of untreated GD for both mother and fetus [11]. The premise of the search for early markers of GD severity is that it may be useful to predict which patients will develop more severe forms of metabolic alterations to improve the standard of care through early intervention, closer monitoring, and personalized treatment. This would lead to optimization of maternal and perinatal outcomes [12, 13].

The aim of our study was to evaluate the longitudinal changes of the plasma lipid profile in patients diagnosed as affected by gestational diabetes according to the International Association of Diabetes and Pregnancy Study Group (IASGDP) criteria [14] and treated according to the standard management and therapy in order to assess potential markers of disease severity especially in case of the need for pharmacological therapy to control glycemia. To this aim, the plasma FA lipidomic analysis was performed in association with markers of inflammation and oxidative stress related to the clinical outcomes.

## Materials and methods

### Study design and population

We conducted a single-centre randomised placebo-controlled double-blind study at Fondazione IRCCS Ca' Granda Ospedale Maggiore Policlinico, Milan, Italy (Mangiagalli High Risk Maternity Centre). The study was conducted from May 2018 to March 2020, and approved by the Milan Area 2 Ethics Committee (PRE.D.I.P.2, project identification code 4004, approval number 126 on 28 March 2018). Women were recruited during their first obstetrical visit at maternal–fetal medicine outpatient clinics, and patients who had obtained a positive result on the oral glucose tolerance test with 75 g glucose between 24 and 28 weeks of gestation (according to the recommendations of the IADPSG [14]) were included. Written informed consent was obtained from all enrolled patients. The following were excluded: multiple pregnancies, pregnant women under 18 years of age, women with evidence of fetal malformations, and chronic ma-ternal conditions (type 1 and 2 diabetes, hypothyroidism and hyperthyroidism, immunological disorders).

During the first study visit at 24–28 weeks, at recruitment, women were randomized to receive either anti-inflammatory dietary supplements (intervention group) or placebo (control group). The anti-inflammatory supplements included the following: Omega-3 fatty acids (tablets, EnerZona Omega3Rx®, Enervit, Italia), at a daily dosage of 2.4 gr at breakfast; anthocyanins (tablets, EnerZona Maqui Response Capsule®, Enervit, Italia) at a total daily dose of 108 mg divided into three equal doses at breakfast, lunch, and dinner. Details of the supplementation protocols and results have been published previously [5].

All participants were instructed to self-monitor capillary blood glucose levels (fasting and 2-h postprandial measurements) three times per day using a standard reflectance meter; recordings of these values were reviewed at each antenatal visit.

Patients received ultrasonography for fetal growth every two weeks, evaluated according to the international standards from the Intergrowth-21st project [15]; estimated fetal weight was calculated according to Hadlock formula [16]. Patients also received weekly dietary advice, and fetal wellbeing assessments at term. The final study visit took place after at least 12 weeks of treatment at 36–39 weeks of gestation, then patients were monitored for maternal and fetal well-being until delivery, according to the protocol used at our institute.

Personalized insulin regimens were prescribed if mean blood glucose levels were elevated in relation to fetal growth as assessed by ultrasound (fasting glucose level ≥ 95 mg/dL or 2-h postprandial glucose level ≥ 120 mg/dL if the fetal abdominal circumference was below the 75th percentile for gestational age; fasting glucose level ≥ 90 mg/dL or 2-h postprandial glucose level ≥ 110 mg/dL if the fetal abdominal circumference was at or above the 75th percentile for gestational age).

### Monitoring

Baseline characteristics, pregnancy, and neonatal outcomes were recorded for all randomized women. Fasting blood and urine samples were collected from all patients at recruitment and at the final study visit for measurement of metabolic, inflammatory, antioxidant parameters, and plasma fatty acid composition.

### Variables

We performed a quali- and quantitative analysis of total plasma esterified fatty acids. To this aim, plasma was separated from heparinized blood by centrifugation (2000 rpm for 10 min) and stored at − 80 °C until analysis. Plasma esterified fatty acids were determined by gas-chromatography analysis after derivatization with sodium methoxide in methanol [17]. For quantitative determination, samples were spiked with internal standard (TAG C17:0); fatty acid methyl ester (FAME) standards (Sigma-Aldrich, St. Louis, MO, USA) were used for calibration. Gas-chromatography was performed with Shimadzu GC-2025 equipped with flame ionization detector (FID). The separation was achieved with capillary Zebron FAME, length 30 m × 0.25 mm I.D., film thickness 0.20 µm; carrier gas, helium; injector temperature, 250 °C; detector temperature, 275 °C; oven temperature, 100 °C for 2 min and then increased at rate of 10 °C min^−1^ to 250 °C.

Plasma inflammatory markers were measured using commercial ELISA kits using the Biomek 4000 ELISA microplate liquid reagent dispensing automation tool (Beckman Coulter, Brea, CA, USA) and the EL405LS ELISA microplate automated washing system (BioTek Instruments, Winooski, VT, USA) and a multiskan FC plate reader (Thermo Scientific, Waltham, MA, USA). PAF was assayed using Elabscience ELISA Kit (lower range of detection 0.313 ng/mL, sensitivity 0.188 ng/mL, Elabscience, Houston, TX, USA).

Methylglyoxal (MGO) was measured using the OxiSelectTM Methylglyoxal Competitive ELISA Kit (lower range of detection 0 g/mL, Cell Biolabs, San Diego, CA, USA), which is an enzyme immunoassay designed to detect and quantify protein adducts of methylglyoxal-hydro-imidazoline.Serum TNF-α was measured using an Ultrasensitive ELISA Kit (lower range of detection 0 pg/mL, sensitivity < 0.09 pg/mL, Invitrogen, ThermoFisher Scientific, Carlsbad, CA, USA).

BAFF was assayed using the Human BAFF/BLyS/TNFSF13B Immunoassay Quantikine® ELISA (lower range of detection 0 pg/mL sensitivity 2.68 pg/mL, R&D Systems Inc, Minneapolis, MN, USA).

The absorbance of each well was read at a wavelength of 450 nm with a Multiskan FC plate reader (Thermo Scientific, Waltham, MA, USA). The average zero standard optical density was subtracted from all absorbances, and a standard curve was generated using a four-parameter logistic (4-PL) curve fit. The concentration in the test sample was calculated through interpolation along the standard curve by multiplying the result by the dilution factor.

Urinary markers of oxidative stress were measured in urine using commercially available ELISA kits, according to the manufacturer instructions.

8-isoprostane was determined using Cayman’s ELISA Kit (detection range 0.8–500 pg/ml; sensitivity of approximately 3 pg/ml, 80% B/B0; Cayman Chemicals, Cedarlane Labs, Canada).

8-OHdG, 8-OHG, and 8-hydroxyguanine were determined using Cayman's DNA/RNA Oxidative Damage (High Sensitivity) ELISA Kit (detection range 10.3–3000 pg/ml; sensitivity of approximately 30 pg/ml, 80% B/B0; Cayman Chemicals, Cedarlane Labs, Canada).

The absorbance of each well was read at a wavelength of 405 nm with a Tecan Infinite F500 plate reader (TECAN, Milano, Italia). The average zero standard optical density was subtracted from all absorbances, and a standard curve was generated using eight-parameter logistic curve fit. The concentration in the test sample was calculated through interpolation along the standard curve by multiplying the result by the dilution factor.

### Statistical analysis

Statistical analysis of the data was performed using GraphPad Prism 9 for macOS (GraphPad Software, San Diego, CA, USA. Version 9.3.1 (350)). The median and interquartile range (IQR) were calculated for each variable. The medians were compared using the Mann–Whitney test. The Chi-square test of association was used to evaluate the relationships between categorical variables. A p-value < 0.05 was used as the limit of statistical significance.

## Results

We recruited 51 women, of whom 40 completed the protocol. As previously described, no differences were noted between the intervention and placebo groups (see Materials and Methods) concerning blood and urine measurements of metabolic, inflammatory, antioxidant, and fatty acid parameters [5]. Furthermore, data on anthropometric and routine laboratory parameters—including glycaemia, HbA1c, insulin, total cholesterol, LDL cholesterol, HDL cholesterol, triglycerides, C-reactive protein, and cortisol—were previously published [5]. At baseline (T0), no significant differences were found between the intervention (IG) and placebo (PG) groups regarding anthropometric, metabolic, and inflammatory parameters. The mean age was comparable between IG (34 years, IQR 32–37) and PG (34 years, IQR 33–38). Height also showed no significant differences, with a median of 161 cm (IQR 159–165) in IG and 163 cm (IQR 160–167) in PG. Pre-pregnancy weight and BMI were similar, with IG reporting a weight of 58.8 kg (IQR 51.5–66.0) and a BMI of 22.4 kg/m^2^ (IQR 20.2–23.7), while PG recorded a weight of 62.0 kg (IQR 56.5–68.5) and a BMI of 23.3 kg/m^2^ (IQR 21.3–24.7). Regarding glycaemic indices, fasting glycaemia was 73 mg/dL (IQR 67–77) in IG and 72 mg/dL (IQR 66–75) in PG, while HbA1c levels were 31 mmol/mol (IQR 30–32) and 30 mmol/mol (IQR 28–31), respectively. Insulin levels were comparable between the two groups (IG: 7.1 µU/mL, IQR 5.2–14.2; PG: 9.5 µU/mL, IQR 6.2–14.3). Lipid profile analysis revealed no significant differences in total cholesterol (IG: 265 mg/dL, IQR 226–279; PG: 230 mg/dL, IQR 218–275), LDL cholesterol (IG: 122 mg/dL, IQR 103–152; PG: 146 mg/dL, IQR 144–188), HDL cholesterol (IG: 73 mg/dL, IQR 64–85; PG: 81 mg/dL, IQR 65–88), and triglycerides (IG: 182 mg/dL, IQR 128–213; PG: 175 mg/dL, IQR 148–211). After 12 weeks of treatment (T12), no significant differences between groups were recorded in anthropometric, metabolic, or inflammatory parameters. Arm circumference remained similar between IG (28.6 cm, IQR 26.8–29.3) and PG (28.5 cm, IQR 28.0–30.0), as did wrist circumference (IG: 15 cm, IQR 14.5–15.5; PG: 15 cm, IQR 14.5–16.0) and waist circumference (IG: 94.5 cm, IQR 91–99; PG: 97 cm, IQR 93–102). Skinfold measurements, including bicipital (IG: 8.5 mm, IQR 7.3–10.3; PG: 10.4 mm, IQR 7.7–15.8), triceps (IG: 20.0 mm, IQR 19.0–23.4; PG: 23.8 mm, IQR 18.4–27.3), and subscapular (IG: 15.8 mm, IQR 14.4–19.0; PG: 16.4 mm, IQR 14.4–24.0), also did not differ significantly. Glycaemic control remained stable in both groups, with fasting glycaemia of 72 mg/dL (IQR 68–82) in IG and 71 mg/dL (IQR 66–78) in PG. HbA1c levels were 33 mmol/mol (IQR 32–36) in IG and 33 mmol/mol (IQR 31–34) in PG. Insulin levels showed no variation, remaining at 9.7 µU/mL (IQR 7.4–14.9) in IG and 9.3 µU/mL (IQR 5.8–15.1) in PG. Lipid profile measurements at T12 were consistent between groups, with total cholesterol at 269 mg/dL (IQR 228–313) in IG and 250 mg/dL (IQR 221–278) in PG. LDL cholesterol levels were 146 mg/dL (IQR 114–188) in IG and 126 mg/dL (IQR 106–154) in PG, while HDL cholesterol remained at 69 mg/dL (IQR 57–80) in IG and 73 mg/dL (IQR 65–87) in PG. Triglyceride levels were stable at 229 mg/dL (IQR 181–271) in IG and 227 mg/dL (IQR 195–283) in PG. Moreover, maternal and neonatal outcomes did not differ between groups. The birth weight was 3250 g (IQR 3073–3428) in IG and 3215 g (IQR 2933–3050) in PG. Similarly, the birth weight percentile remained at 34 (IQR 20–64) in IG and 33 (IQR 12–67) in PG. Overall, these findings indicate that after 12 weeks of treatment, there were no statistically significant differences between the intervention and placebo groups in anthropometric, metabolic, or inflammatory parameters, or maternal or neonatal outcomes.To evaluate the impact of GD on the plasma lipidomic profile we measured plasma esterified fatty acids in all the enrolled women comparing the time of diagnosis (T0) with 12 weeks later (T12) (Table 1).

**Table 1:** Plasma fatty acid composition in enrolled patients affected by gestational diabetes

	T0 (n = 51)		T12 (n = 40)		Δ	
	μg/mlplasma	% of totallipids	μg/mlplasma	% of totallipids	μg/ml	p
C16:0	750 (630–875)	30.0 (28.1–31.6)	888 (741–1029)	30.2 (27.6–33.0)	138.5	0.010
C16:1	48 (39–71)	2.0 (1.6–2.5)	65 (47–93)	2.1 (1.9–2.7)	16.5	0.029
C18:0	130 (106–150)	5.0 (4.5–5.7)	135 (119–186)	4.8 (4.6–5.6)	4.7	0.339
C18:1	576 (497–731)	22.8 (21.4–25.3)	690 (585–862)	23.6 (22.1–27.1)	114.7	0.005
C18:2	643 (545–716)	24.3 (21.0–27.9)	681 (591–877)	24.8 (22.5–26.8)	37.6	0.017
C18:3 n-6	5 (3–7)	0.2 (0.1–0.3)	6 (4–10)	0.2 (0.1–0.3)	0.9	0.426
C18:3 n-3	7 (5–14)	0.3 (0.2–0.5)	8 (6–20)	0.3 (0.2–0.7)	0.6	0.103
C20:3	37 (26–50)	1.4 (1.1–1.8)	34 (27–45)	1.2 (1.0–1.5)	-2.4	0.874
C20:4	218 (180–277)	8.7 (7.5–9.7)	188 (159–278)	6.7 (5.9–7.9)	-29.5	0.494
C20:5	12 (7–17)	0.4 (0.3–0.7)	13 (7–20)	0.4 (0.2–0.8)	1.1	0.235
C22:5	7 (6–11)	0.3 (0.2–0.4)	8 (5–13)	0.3 (0.2–0.4)	0.3	0.767
C22:6	81 (63–101)	3.2 (2.7–3.9)	78 (65–95)	2.6 (2.1–3.4)	-3.0	0.837
SFA	888 (737–1049)	34.9 (33.7–36.5)	1021 (868–1165)	34.7 (33.2–38.0)	133.7	0.013
MUFA	644 (542–793)	25.3 (23.2–27.5)	752 (625–965)	26.3 (24.6–28.6)	108.5	0.004
PUFA	1052 (869–1165)	39.0 (36.6–42.7)	1032 (873–1264)	37.0 (33.5–41.2)	-20.6	0.106
n-6 PUFA	911 (746–1028)	34.5 (32.1–38-4)	907 (777–1149)	33.3 (30.2–36.3)	-3.6	0.090
n-3 PUFA	117 (89–137)	4.5 (3.6–5.3)	109 (86–157)	3.9 (3.1–5.0)	-8.8	0.482
Total FA	2575 (2202–3049)		2803 (2520–3335)		227.7	0.013

T0 = time at the diagnosis of gestational diabetes; T12 = time after 12 weeks from the diagnosis; C16:0 = Palmitic acid; C16:1 = Palmitoleic acid; C18:0 = Stearic acid; C18:1 = Oleic acid; C18:2 = Linoleic acid; C18:3 n-6 = gamma-Linolenic acid; C18:3 n-3 = alpha-Linolenic acid; C20:3 =Dihomo-gamma-linolenic acid; C20:4 = Arachidonic acid; C20:5 = Eicosapentaenoic acid; C22:5 = Docosapentaenoic acid; C22:5 = Docosahexaenoic acid; *SFA* saturated fatty acids; *MUFA* monounsaturated fatty acids; *PUFA* polyunsaturated fatty acids; n-6 PUFA = omega 6 polyunsaturated fatty acids; n-3 PUFA = omega 3 polyunsaturated fatty acids; *FA* fatty acids. The p-value refers to the concentration expressed in μg/ml

During pregnancy, in GD women there is a statistically significant increase in total plasma FAs, from 2575 (2202–3049) μg/ml of plasma in the second trimester of gestation to 2803 (2520–3335) μg/ml of plasma at the end of the third trimester (p = 0.013). In particular, the increase in total plasma FAs is due to an increase in palmitic acid (C16:0), palmitoleic acid (C16:1), oleic acid (C18:1) and linoleic acid (C18:2) (Table 1).

Subsequently, we performed a subgroup analysis comparing the women who did not require pharmacological therapy to regulate blood glucose levels (GD group; n = 45 at T0, n = 34 at T12) with those who required additional insulin as pharmacological therapy (GD + PT group; n = 6 at T0 and T12). Comparing the GD group and the GD + PT group 12 weeks after diagnosis, the glycemic compensation, which was already altered at the time of diagnosis as described previously [5], was still partially altered despite supportive pharmacological therapy.

**Fig. 1:**
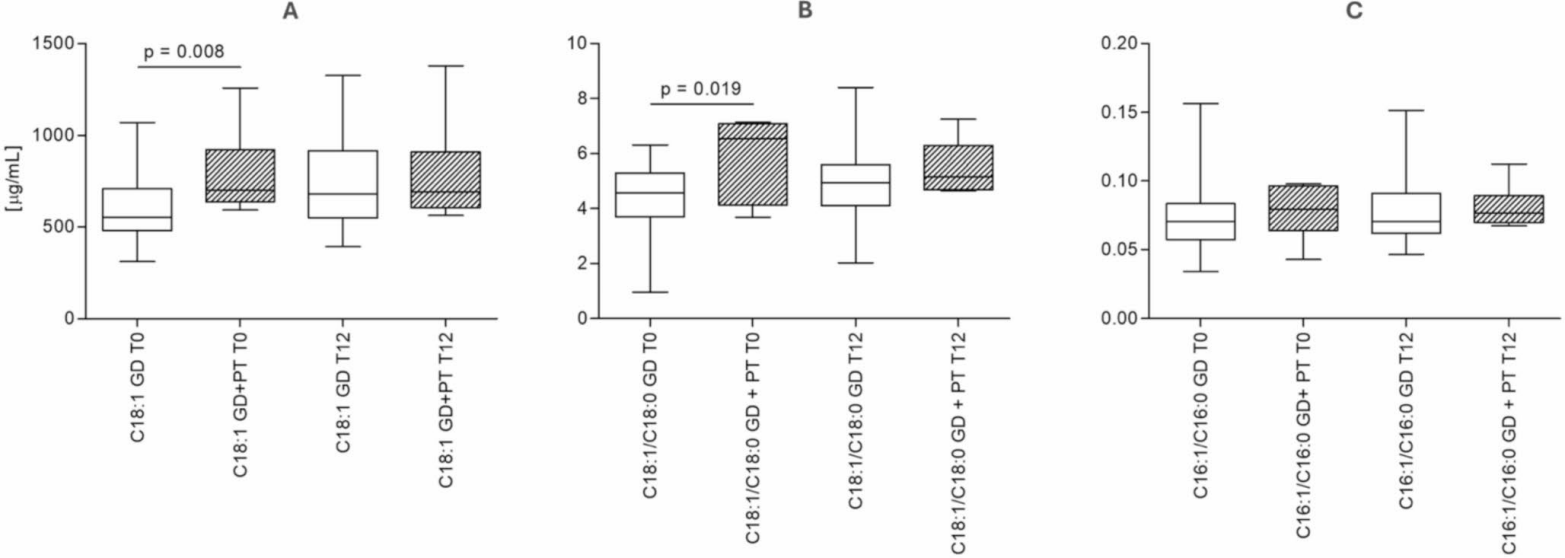
Oleic acid (C18:1n-9) concentration (**A**), oleic acid/stearic acid ratio (18:1n-9/18:0) (**B**) and palmitoleic acid/palmitic acid ratio (16:1n-7/16:0) (**C**) at T0 and T12 in women with gestational diabetes who did not require pharmacological intervention (GD; n = 45 at T0, n = 34 at T12) and in patients with gestational diabetes who required therapy to normalize blood glucose levels (GD + PT; n = 6)

At the time of diagnosis (T0), GD group patients compared to those of GD + PT group showed a statistical difference in the plasma concentration of C18:1 [552 (IQR 479–709) µg/ml and 700 (IQR 637–921) µg/ml p = 0.008, respectively]. However, this difference was no longer detectable 12 weeks after diagnosis [679 (IQR 551–916) µg/ml and 692 (IQR 606–909) µg/ml] (Fig. 1A).

The desaturation index used as an estimate of SCD-1 activity showed higher levels of desaturation at T0 and T12 in the GD + PT patients in the oleic acid/stearic acid ratio (18:1n-9/18:0) [6.54 (4.13–7.08) for GD + PT and 4.56 (3.70–5.29) for GD at T0 (p = 0.0193); 5.17 (4.68–6.30) for GD + PT and 4.93 (4.10–5.60) for GD at T12 (p = 0.264)] (Fig. 1B). In contrast, the palmitoleic acid/palmitic acid ratio (16:1n-7/16:0) showed no statistically significant differences [0.079 (0.064–0.096) for GD + PT and 0.071 (0.058–0.084) for GD at T0 (p = 0.359); 0.077 (0.070–0.090) for GD + PT and 0.071 (0.062–0.091) for GD at T12 (p = 0.373)] (Fig. 1C). There were no other statistically significant differences in other plasma FAs between the two groups.

Comparison of these two groups of GD patients revealed a difference in methylglyoxal (MGO) concentration, both at T0 and T12, which appeared to be higher in GD + PD group (Table 2). There were no other statistically significant differences in plasma inflammatory and urinary oxidative stress parameters (Table 2).

**Table 2:** Inflammatory parameters at the time of diagnosis of gestational diabetes and after 12 weeks in women who did not require pharmacological intervention (GD) and in patients who required therapy to normalize blood glucose levels (GD + PT)

	T0			T12		
	GD (n = 45)	GD + PT (n = 6)	p	GD (n = 34)	GD + PT (n = 6)	p
Urine 8-isoprostane (pg/mL)	1155 (653–1531)	0.591 (0.450–0.754)	0.183	770 (499–1250)	887 (642–1189)	0.892
Urine DNA/RNA oxidative damage (pg/mL)	4993 (3162–8168)	6294 (3247–8897)	0.787	5310 (2556–8246)	6654 (3247–8897)	0.430
Plasma BAFF (ng/mL)	1.23 (1.07–1.37)	1.16 (1.06–1.38)	0.861	1.29 (1.05–1.48)	6654 (3247–8897)	0.710
Plasma PAF (ng/mL)	31.3 (19.0–49.4)	26.7 (17.1–38.5)	0.689	39.4 (30.5–73.4)	39.7 (23.5–64.4)	0.783
Plasma TNF-α (pg/mL)	31.3 (19.0–49.4)	3.31 (3.03–3.58)	0.529	3.50 (3.10–3.70)	3.72 (3.38–4.07)	0.724
Plasma MGO (μg/mL)	0.591 (0.450–0.754)	0.976 (0.927–1.181)	0.001	0.665 (0.455–0.871)	1.126 (1.001–1.226)	0.014

*T0* time at the diagnosis of gestational diabetes; *T12* time after12 weeks from the diagnosis; *BAFF* B-cell activating factor; *PAF* platelet activating factor; *TNF-α* tumor necrosis factor-alpha; *MGO* methylglyoxal

**Fig. 2:**
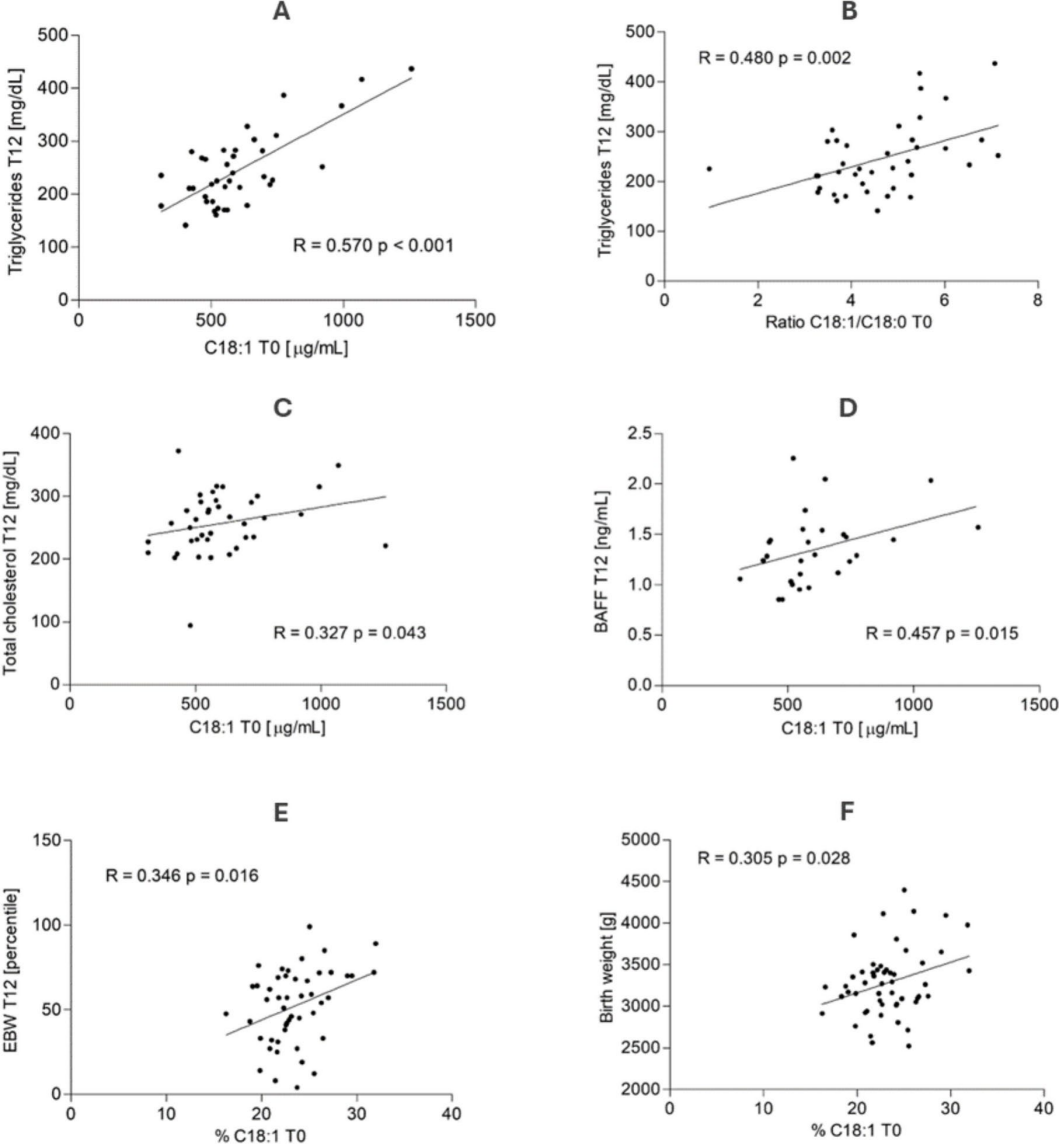
Correlation between the concentration of plasma oleic acid (C18:1 n-9) expressed in μg/mL at diagnosis of gestational diabetes (T0) and the concentration of triglycerides (**A**), total cholesterol (**C**) and BAFF (**D**) 12 weeks after diagnosis (T12). Correlation between the C18:1/C18:0 ratio at T0 and triglyceride concentration at T12 (**B**). Correlation between C18:1 concentration at T0 expressed as a percentage of total lipids and estimated birth weight at T12 expressed in percentiles (**E**) and birth weight (**F**). (*EBW* estimated birth weight; *BAFF* B-cell activating factor)

To understand if the oleic acid can represent a prognostic value for the pharmacological treatment of GD, we considered the correlation between the concentration of plasma C18:1 at T0 and other metabolic markers and anthropometric parameters at 36–39 weeks of gestation (T12). Figure 2 depicts the more relevant correlations.

A positive correlation was found with the concentration of TAGs at T12 (R = 0.570 p < 0.001, Fig. 2A and also with total cholesterol (R = 0.327 p = 0.043, Fig. 2C). A similar positive correlation also existed between the oleic/stearic fatty acid ratio (18:1n-9/18:0) and the TAG concentration at T12 (R = 0.480 p = 0.002, Fig. 2B).

In relation to inflammatory markers, a positive correlation was detected with the concentration of BAFF at T12 (R = 0.457 p = 0.0145, Fig. 2D).

The analysis of fetal anthropometric measurements revealed significant differences between the study groups, with variations in the percentiles of abdominal circumference (AC), cranial circumference (CC), femur length (FL), and estimated birth weight (EBW) at T0, as well as differences in AC and EBW at T12. These differences were observed explicitly between patients who required pharmacological therapy to normalise blood glucose levels and those who did not. Further details on specific values are provided in Table 3. Notably, the concentration of C18:1 was already positively correlated with the fetal anthropometry measured at T0, particularly with the infant's AC (R = 0.303, p = 0.029), the infant's CC (R = 0.300, p = 0.031), and with the EBW expressed as a percentile according to gestational age (R = 0.376, p = 0.006). When the concentration of C18:1 was expressed as a percentage of the total lipids, the positive correlation was confirmed with EBW at T12 (R = 0.346, p = 0.016, Fig. 2E) and with the infant's birth weight (R = 0.305, p = 0.028, Fig. 2F).

**Table 3:** Ultrasound fetal measurements at gestational diabetes diagnosis and after 12 weeks in women who did not need pharmacological treatment (GD) compared to those who required therapy to regulate their blood glucose levels (GD + PT)

	T0			T12		
	GD (n = 45)	GD + PT (n = 6)	p	GD (n = 34)	GD + PT (n = 6)	p
AC (percentile)	50.8 (38.9–64.1)	67.8 (59.5–82.3)	0.009	55.0 (36.3–76.4)	83.0 (72.8–91.5)	0.027
BPD (percentile)	60.8 (43.8–82-3)	70.0 (63.5–72.4)	0.631	52.0 (31.9–70.7)	62.0 (45.5–90.5)	0.223
CC (percentile)	47.1 (28.7–58.8)	69.0 (59.0–73.5)	0.021	35 (19.0–50.0)	44 (21.5–82)	0.136
FL (percentile)	53.7 (46.0–72.6)	70.0 (68.5–88.5)	0.036	49.6 (32.8–62.0)	59 (49.5–75.5)	0.321
EBW (percentile)	54.6 (41.9–61.5)	80 (71.5–82.5)	< 0.001	51 (33.0–64.0)	72 (71.0–82.5)	0.002

T0 = time at the diagnosis of gestational diabetes; T12 = time after 12 weeks from the diagnosis; *AC* abdominal circumference; *BTD* = biparietal diameter; *CC* cranial circumference; *FL* femur length; *EBW* estimated birth weight

## Discussion

In terms of predicting GD, studies have focused on glucose metabolism and the association between elevated levels of glycated hemoglobin, insulin, HOMA index and C peptide in the first half of pregnancy and the risk of developing GD [18–21]. Recently, lipidomic analysis in pregnant patients has been also explored in terms of predicting GD [9], and it has been found that impaired lipid homeostasis is present years before the onset of diabetes in women with GD [22, 23]. Also, specific FAs play different roles in the regulation of glucose homeostasis before the diagnosis of GD [8].

In our study, we performed a lipidomic assessment of patients at GD diagnosis (24–28 weeks of gestation) and 12 weeks later. Although in a small group of patients, our data clearly indicates that a further worsening of clinical conditions of gestational diabetes requiring medical therapy is characterized by increased levels of maternal plasmatic lipids. In particular, we found higher levels of plasma esterified oleic acid at the time of GD diagnosis in pregnant patients who later required pharmacological therapy to control blood glucose levels compared to those who did not require supplementary treatment. No differences were observed for the other FAs analyzed.

Changes in lipidomics, observed after GD treatment despite the lowering of blood glucose levels [24, 25], may partially explain the cases of LGA infants found in well-controlled diabetes [26, 27], and are consistent with Freinkel’s theory of fetal overgrowth due to multiple nutritional factors [3]. Maternal plasma lipids correlate with neonatal weight and adiposity in the presence of adequate glycemic control [28]. Accordingly, our study found a correlation between lipid profile, particularly plasma oleic acid concentrations, and fetal growth, in the context of adequate glycemic control, supporting the hypothesis that women with GD are affected by a metabolic dysregulation involving not only the glucose metabolism but also other pathways, the identification of which could help clinicians to discriminate different cases within the spectrum of severity of the disease. Our study focused on the metabolic determinants of fetal growth rather than precisely predicting LGA. Rather than adopting a binary classification, we opted for a linear regression approach to explore the continuous relationship between maternal lipidomic profiles and fetal anthropometry. Since we also had access to neonatal birth weight, we prioritized an analysis that could provide broader insights into fetal growth dynamics. However, we acknowledge that alternative predictive approaches, such as logistic regression or machine learning, could be valuable for future studies focused on LGA risk stratification. At the state of art, it is not possible to assign to a specific compartment the increase of oleic acid and TAGs in the maternal plasma. It’s reasonable to hypothesize that maternal insulin resistance is responsible for the greatest part of these circulating lipid fuels, but at the same time it cannot be excluded that the placenta may contribute to these increased levels, especially in conditions of worsen insulin resistance, when medical therapy is required. Although insulin cannot cross the placenta, its specific receptor is present in the trophoblast membrane, activating the specific insulin pathways of placental metabolism of fuels. Although it is well known that the physiological insulin resistance to peripheral tissues is worsened by conditions as GD, the grade of severity of insulin resistance of this disease is not completely studied and understood inside the placenta. It has been clarified that GD is characterized by a decrease in the expression of lipoprotein lipase (LPL), whose reduction has been explained at the light of a potential mechanism to reduce the excess of maternal transfer of fatty acids to the fetus [29]. On the other hand, the decreased activity of LPL might increase the storage of lipids inside the trophoblast creating in a certain kind of placental steatosis a reservoir to direct again the lipids toward the maternal compartment. This further increase of lipids might facilitate the availability in other tissues, as the maternal liver exacerbating the insulin resistance in a vicious circle.

Altered lipid profiles affect the metabolic physiology of pregnancy through various pathological mechanisms. Changes in lipid transporter expression and fatty acid-binding proteins 4 (FABP4), which intracellularly bind FAs, impact on gene transcription, inflammation, and intracellular signaling through different pathways, including IKK/NFkB [30]. In addition, inflammatory mediators, such as BAFF, have been described to be involved in the regulation of body weight and the response to increased levels of circulating FFAs as induced by a high-fat obesogenic diet [31, 32]. On the other hand, higher concentration of circulating esterified FAs, such as oleic acid, can also be induced by increased endogenous production and, possibly, by greater placental production. The correlation between oleic acid concentration and circulating BAFF levels at the time of diagnosis and at 12 weeks adds a possible mechanism to support the pro-inflammatory and pro-diabetic state in the metabolic set of GD. Numerous studies have documented that BAFF can directly stimulate an increase in insulin resistance [33], providing a possible link between increased levels of inflammation, altered glycemic metabolism and weight gain by modulating the production of other inflammatory cytokines, such as TNF, IL-6 and PAF, and amplifying the signal between adipocytes and inflammatory cells that can lead to obesity [34]. Higher levels of PAF and TNF-α have been found in GD patients with good glycemic control and their increase during pregnancy has been proposed as an additional tool to monitor this condition [35]. The increase in MGO found in GD + PT patients compared to those in GD group indicates an alteration in glycemic metabolism. The production of MGO is promoted by hyperglycemia and can lead to an increase in the production of advanced glycation end-products (AGEs) and an increase in lipid peroxidation as well as altering mitochondrial function [36–38]. As suggested [35], MGO could be interpreted as another early marker of the severity of GD.

From a nutritional point of view, the Mediterranean diet, rich in olive oil and, therefore, in oleic acid, has been considered a healthy dietary pattern associated with a reduction in cardiovascular and metabolic diseases. The health benefits were initially attributed to the content of MUFA, but nowadays, the content of minor compounds, such as polyphenols, and the diversity of Mediterranean diet composition are considered to play a synergistic role in this contest [39]. On the other hand, there is evidence that the use of dietary oleic acid (i.e. olive oil) reducing SFAs may be the best nutritional and therapeutic approach for the treatment of insulin resistance and type 2 diabetes mellitus [40].

Worth of note, different lipid fractions in human plasma have characteristic FA profiles which are partially maintained by diet and through endogenous synthesis. In fact, oleic acid is largely synthesized in our body, mainly in the liver and adipose tissue, esterified in TAGs, and distributed in plasma to other tissues via low- or very-low-density lipoproteins (LDLs/VLDLs). In peripheral tissues lipids are imported by specific transmembrane transporters, such as FABPs and CD36 FA translocase, as well as members of the FA transport proteins (FATP1–6) and solute carrier family. SCD-1 is the key enzyme involved in the endogenous synthesis of MUFAs, and its expression and activity are increased in several types of diseases including diabetes and cancer [41]. SCD-1 is tightly controlled at the transcriptional and post-translational levels by dietary and hormonal signals with induction by carbohydrate, fat, and insulin and suppression by PUFA and leptin [42, 43].

While studies in adult humans have shown that plasma reflects hepatic SCD-1 expression, this has not been confirmed in fetal life. Preliminary studies have hypothesized that exposure to a changed uterine nutrient environment in GD may upregulate placental and fetal SCD-1, as suggested by the increased desaturation index in umbilical cord plasma, which in turn may correlate with infant adiposity [44]. FA transport across the placenta contributes to determining the composition of the fetal body through endogenous metabolism but is not fully understood. Long-chain PUFAs are favored for delivery to the fetus. Cellular uptake and intracellular translocation of non-esterified FAs have been proposed as a multistep process facilitated by various membrane-associated and cytoplasmic proteins, such as lipoprotein lipase and FABPs which may drive the bidirectional flux of FAs [45].

The limited size of the two groups is the main limitation of our work. Further studies with a larger cohort are needed to confirm our results. In addition, our study population was mainly represented by Caucasian women due to the usual referrals to the hospital involved. Blood glucose control was evaluated through patients’ diaries and was not double checked on glucometers.

## Conclusions

Considering these complex relationships and the controversial role of SCD-1 and oleic acid in health and insulin resistance, we hypothesized that adverse maternal conditions in GD may stimulate maternal and fetal SCD-1 activity and thus influence neonatal growth. A complex relationship between maternal, placental and fetal tissues capable of synthesizing oleic acid may regulate SCD-1 activity and insulin sensitivity, resulting in an increased plasma oleic acid. Oleic acid might be suggested as an appropriate tool for the early identification of patients with a more severe form of GD and to serve as an early marker for stratifying the severity of GD, although its measurement is not standardized and is more expensive than measuring blood glucose. This marker could provide early detection and indicate an alteration preceding glycemic decompensation, even when evaluated with a sensor that continuously monitors blood glucose. Therefore, it is an intriguing prospect for understanding the pathogenetic mechanisms underlying GD and its potential fetal consequences.
